# Stronger Response to the Aerosol Indirect Effect Due To Cooling in Remote Regions

**DOI:** 10.1029/2022GL101184

**Published:** 2022-10-31

**Authors:** Linnea Huusko, Angshuman Modak, Thorsten Mauritsen

**Affiliations:** ^1^ Department of Meteorology Stockholm University Stockholm Sweden; ^2^ Now at Interdisciplinary Programme in Climate Studies Indian Institute of Technology Bombay Mumbai India

**Keywords:** aerosol‐cloud interaction, aerosol indirect effect, forcing efficacy

## Abstract

It is often assumed that effective radiative forcings, regardless of forcing agent, are additive in the temperature change. Using climate model simulations with abruptly applied aerosol forcing we find that the temperature response per unit forcing is larger if induced by aerosol‐cloud interactions than directly by aerosols. The spatial patterns of forcing and temperature change show that aerosol‐cloud interactions induce cooling over remote oceans in the extratropics, whereas the effect of increased emissions is localized around the emission sources primarily over tropical land. The results are consistent with ideas of how the patterns of sea surface temperature impact radiative feedbacks, and a large forcing efficacy of aerosol‐cloud interactions could help explain previously observed intermodel spread in the response to aerosols.

## Key Points

- The forcing efficacy from an enhanced aerosol indirect effect is larger than unity
- The aerosol indirect effect induces remote cooling at mid‐ to high‐latitudes, in contrast to the local cooling from the direct effect
- The different spatial patterns of temperature change from the aerosol direct and indirect effects excite different feedbacks

## 1 Introduction

The state of the climate system is determined by the radiative balance at the top of the atmosphere: a positive imbalance causes warming of the system, and vice versa. The largest contributor of uncertainty to the total imbalance is the radiative effect from anthropogenic aerosols, particularly from aerosol‐cloud interactions (Forster et al., 2021). When studying the temperature response to an applied radiative forcing a linear energy balance framework is often used, where the global mean top of atmosphere (TOA) radiative imbalance, *N*, is a function of an external effective radiative forcing, *F*, and the resulting surface air temperature change, Δ*T* (relative to an unforced reference state), according to

(1) N=F+λΔT,

where λ is a feedback parameter (Gregory et al., 2004). It is usually assumed that the feedback parameter, λ, is universal, such that the individual effective radiative forcings that make up *F* can be added linearly without scaling. This assumption is used by the Intergovernmental Panel of Climate Change (IPCC) in their Sixth Assessment Report (Forster et al., 2021), among other things to make projections of global warming in the 21st Century.

It has, however, been shown that in global climate models the temperature response per unit forcing varies depending on the type of forcing, both in idealized scenarios with abruptly applied forcing (e.g., Hansen et al., 2005; Modak & Bala, 2019; Modak et al., 2016, 2018; Richardson et al., 2019; Shindell et al., 2015) and in transient scenarios (e.g., Zhao et al., 2019). The concept of forcing efficacy has been introduced to account for these differences in the temperature response (Hansen et al., 2005). Several studies have argued that the intermodel variation in the efficacy of different forcing agents likely causes discrepancies in estimates of the equilibrium climate sensitivity, defined as the equilibrium temperature response to doubled CO_2_ concentration over preindustrial levels, between model based estimates and constraints from observational data (Kummer & Dessler, 2014; Marvel et al., 2016; Richardson et al., 2019). These studies have highlighted the importance of including forcing efficacy in the linear energy balance framework.

Forcing efficacy can be incorporated into the linear framework as a factor *E*, using CO_2_ as a reference:

(2) $$E=\frac{\lambda_{2\times CO_{2}}}{\lambda },$$

where $\lambda_{2\times CO_{2}}$ is the feedback parameter in a simulation with abruptly doubled atmospheric CO_2_ concentration. Under the assumption of a time‐invariant λ the efficacy can be approximated as

(3) $$E=\frac{\Delta T/F}{\Delta T_{2\times CO_{2}}/F_{2\times CO_{2}}},$$

where Δ*T* and *F* are defined as in Equation 1, and $\Delta T_{2\times CO_{2}}$ and $F_{2\times CO_{2}}$ are the corresponding quantities under a doubling of the CO_2_ concentration. This approximation is used by Richardson et al. (2019) and is adopted here.

The concept of forcing efficacy is not new, yet the aerosol forcing efficacy remains difficult to constrain. Richardson et al. (2019) studied forcing efficacy in a set of global climate models and found a considerable spread in the response to aerosol forcing. Nevertheless, recent studies based on the models that participated in the sixth phase of the Coupled Model Intercomparison Project show an enhanced climate response to aerosol forcing (Salvi et al., 2022; Smith & Forster, 2021). Salvi et al. (2022) suggested that the strong temperature response, compared to the corresponding response to CO_2_ forcing, stems from the latitudinal distribution of the aerosol forcing.

Aerosols affect the radiative balance of the climate system both through direct interaction with radiation (direct effect) and through aerosol‐cloud interactions (indirect effect), for example, by increasing the cloud reflectivity by distributing the cloud water on more abundant but smaller droplets (Twomey effect; Twomey, 1974). However, insight into the relative contributions from the direct and indirect effects from aerosols to the climate response is lacking in the literature. In this study, we systematically investigate the climate response to aerosol forcing by disentangling the aerosol direct and indirect effects. Using MPI‐ESM1.2 we run idealized simulations with aerosol forcing applied abruptly and held constant to assess the climate response. We show that the direct effect causes local cooling and has a forcing efficacy close to unity, while an enhanced indirect effect causes a stronger global mean temperature response per unit forcing. The behavior is related to a remote response at mid‐ to high‐latitudes and consistent with ideas of how the patterns of change influence radiative feedbacks.

## 2 Background: Pattern Effects

How large the temperature response to an applied forcing is depends on the feedback mechanisms in the climate system. The spatial pattern of temperature change is believed to affect the feedback mechanisms that are activated following an initial change in the surface temperature and the idea of so‐called pattern effects has gained much attention (e.g., Armour et al., 2013; Ceppi & Gregory, 2017; Dong et al., 2019, 2020).

Various explanations have been suggested for how pattern effects influence the global climate. Pierrehumbert (1995) pointed to the importance of “radiator fins” over areas with cold sea surface temperatures (SSTs), where subsidence makes the air dry and clear, in stabilizing the climate. Building on this idea, Ceppi and Gregory (2017) argued that changes in tropospheric temperatures aloft govern changes in the global mean feedback parameter with time, and that this is controlled by areas with comparatively warm SSTs, such as the West Pacific warm pool region, where the tropospheric stability depends directly on the local SST. In other regions the stability depends on the SST relative to that in the warmer areas, because heat is advected from warm areas in the free troposphere, leading to temperature inversions over areas with lower SSTs. Using a Green's function approach, Dong et al. (2019) likewise identified temperature change in the tropical West Pacific region as important for the global mean energy balance. A similar effect has been found in studies showing cooling from low clouds forming below inversions (e.g., Mauritsen, 2016; Zhou et al., 2016).

The mechanisms of pattern effects are typically discussed in the context of time‐varying feedbacks under CO_2_ induced warming, but local temperature change due to a localized forcing, such as from aerosols, will cause SST patterns that can be studied analogously. Studies where the climate has been forced with inhomogeneous patterns of carbon dioxide, aerosols, or SSTs suggest that the location of a radiative forcing affects the location and strength of the temperature response (e.g., Dong et al., 2020; Forster et al., 2000; Hansen et al., 1997, 2005; Modak & Bala, 2019; Persad & Caldeira, 2018; Salvi et al., 2022; Stuecker et al., 2020). Here, we investigate the patterns of forcing and the corresponding response to the aerosol direct and indirect effects.

## 3 Model and Methods

We have run simulations with the Max Planck Institute for Meteorology Earth System Model version 1.2 (MPI‐ESM1.2). The next two sections describe the model and the parameterization of the Twomey effect. The third section explains how the experiments were set up.

### 3.1 MPI‐ESM1.2

MPI‐ESM1.2 is a state‐of‐the‐art Earth system model (Mauritsen et al., 2019). The model was used here because it has a simple aerosol scheme (described below) and can be run at a low resolution, and still accurately simulates aerosol forcing close to the best estimate of the IPCC AR5 and a historical global warming in close agreement with observations (Mauritsen et al., 2019). We ran MPI‐ESM1.2 at its lowest resolution (*coarse resolution*, CR; see Mauritsen et al., 2019), as a higher resolution would limit the number of simulated years and thus restrict the comprehensiveness of the analysis. To verify the results from the CR model we also ran a few key experiments in the *low resolution* (LR) version of MPI‐ESM1.2, but it should be noted that the model versions differ in more than just the resolution as some important tuning parameters were also set differently.

### 3.2 A Parameterized Twomey Effect

The complexity of aerosol and cloud interactions makes their climate impact challenging to constrain (Bellouin et al., 2020; Forster et al., 2021). In models with sophisticated interactive aerosol modules the cloud interactions are difficult to isolate and control, whereas in MPI‐ESM1.2, which uses the simple plume implementation of the second version of the Max Planck Institute Aerosol Climatology (MACv2‐SP), this is relatively simple. In this model the aerosol emissions are represented by nine plumes in major source regions, and skewed Gaussian functions are used to represent the spatial distribution of aerosol optical depth (Stevens et al., 2017). In the MACv2‐SP, aerosol‐cloud interactions are represented entirely by the Twomey effect (Stevens et al., 2017; Twomey, 1974). The exclusion of other cloud effects from anthropogenic aerosols, such as the cloud lifetime effect (Albrecht, 1989), was based on the argument that they are too poorly understood (Fiedler et al., 2017). However, here we enhance the Twomey effect as a proxy for representing other uncertain indirect effects. This is reasonable in so far as cloud processes, such as rain formation, are more susceptible to aerosols where also the Twomey effect dominates, that is, where aerosol concentrations are relatively low.

In MACv2‐SP, the strength of the Twomey effect, as described by the cloud droplet number density (*N*), depends on the optical depth of both natural background aerosol (*τ*_*bg*_) and anthropogenic aerosol (*τ*_*a*_), according to

(4) $$\frac{N}{N_{1850}}=\frac{\ln(b_{N}(\tau_{a}+\alpha \tau_{bg})+1)}{\ln(b_{N}\alpha \tau_{bg}+1)},$$

where *b*_*N*_ is a model parameter. The scaling parameter *α* has been introduced here to enable altering of the assumed optical depth of the background aerosol for an enhanced Twomey effect: a reduced background aerosol optical depth (*α* < 1) gives a stronger Twomey effect than with the original formulation.

This simple parameterization of the Twomey effect makes it possible to adjust the strength of the aerosol‐cloud interactions in MPI‐ESM1.2 (Fiedler et al., 2017; Stevens et al., 2017). One could argue that a more complex aerosol module with interactive aerosols would be better for studying the climate response to aerosol‐cloud interactions (e.g., Ekman, 2014), but running a model with an interactive aerosol module is computationally expensive and each change would in principle require a re‐tuning and a new spin‐up of the model (Golaz et al., 2013). The simplicity of MACv2‐SP makes it computationally lightweight and it does not require re‐tuning, yet it produces an evolution of aerosol forcing in line with past estimates (Mauritsen et al., 2019). This allows us to perform a large number of simulations, enabling systematic investigation of the climate response to aerosol forcing of different strengths.

**Figure 1:**
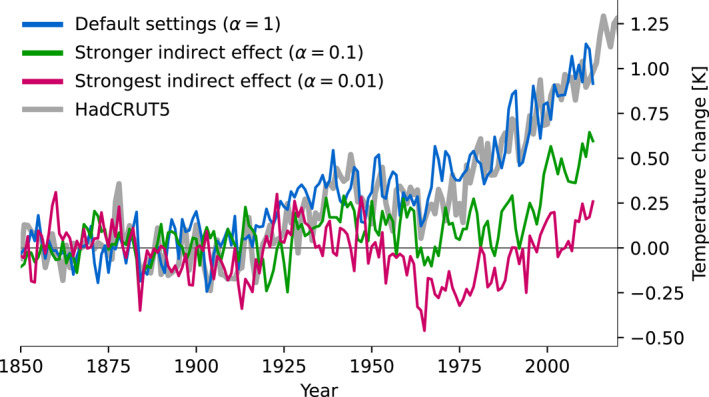
Temperature change over the historical period (1850 to present day) simulated by MPI‐ESM1.2, relative to the 1850–1900 mean. The blue line shows the standard settings in MPI‐ESM1.2 and the green and pink lines show simulations with enhanced aerosol indirect effect (using the scaling parameter *α* in Equation 4). For comparison the figure also includes the HadCRUT5 data set of observed surface temperature over the historical period.

Figure 1 shows simulations of the historical period (1850 to present day) with MPI‐ESM1.2, with standard settings and with an enhanced indirect effect. There is good agreement between the observed warming and the simulated temperature change with the standard setting, in part because the model has been tuned to the observational record (Mauritsen & Roeckner, 2020). An enhanced Twomey effect clearly gives a too cold temperature evolution with the present climate sensitivity; however, different combinations of aerosol cooling and climate sensitivity can be used to achieve a temperature evolution that matches the observational record (e.g., Golaz et al., 2013; Kiehl, 2007).

### 3.3 Experimental Setup

To analyze the climate response to aerosol forcing, idealized simulations were run with forcing applied abruptly and held constant for 150 years. The spatial pattern of aerosol emissions of year 2005 was used in all simulations, and the forcing was strengthened using a combination of enhanced aerosol‐cloud interactions (enhanced indirect effect) and increased aerosol emissions (enhanced direct effect). The experiments with abruptly applied aerosol forcing are here called *abrupt‐aerosol* experiments. For the increased emissions, the 2005 emission levels were scaled by a common factor in all nine emission regions.

Most simulations were run with very strong aerosol forcing. Previous studies looking into different aspects of the forcing and climate response following the shift in the aerosol emission pattern between the 1970s and present day in MPI‐ESM1.2 have found it difficult to distinguish a signal from the internal variability of the model (Fiedler et al., 2017, 2019; Fiedler & Putrasahan, 2021). Therefore, we strongly enhanced the forcing to get a better signal‐to‐noise ratio. Furthermore, to investigate the importance of the location of aerosol emissions we ran additional simulations with emissions from one single region at a time, with emissions from all other plumes turned off.

All simulations were run with a fully coupled model to assess the radiative forcing, temperature response and feedbacks, using the linear regression method suggested by Gregory et al. (2004). Some simulations were also run using only the atmospheric component, ECHAM6.3, with prescribed SSTs fixed in a preindustrial pattern, to obtain the spatial distribution of radiative forcing (Hansen et al., 2005). All coupled runs were 150 years long, while the fixed‐SST simulations were 150 years for the simulations with all emissions and 30 years for simulations with emissions from a single source region. To achieve a stronger signal‐to‐noise ratio, six‐member ensembles were used for some coupled runs. The ensembles were created by running simulations from different initial conditions, selected at 10 year intervals from a preindustrial control simulation.

## 4 Results and Discussion

In the following sections we present the simulated global mean temperature response to aerosol forcing and the implications for forcing efficacy, as well as the spatial patterns of forcing and temperature change. All presented values are anomalies compared to time‐averages from corresponding preindustrial control simulations.

### 4.1 Global Mean Response to Aerosol Forcing

**Figure 2:**
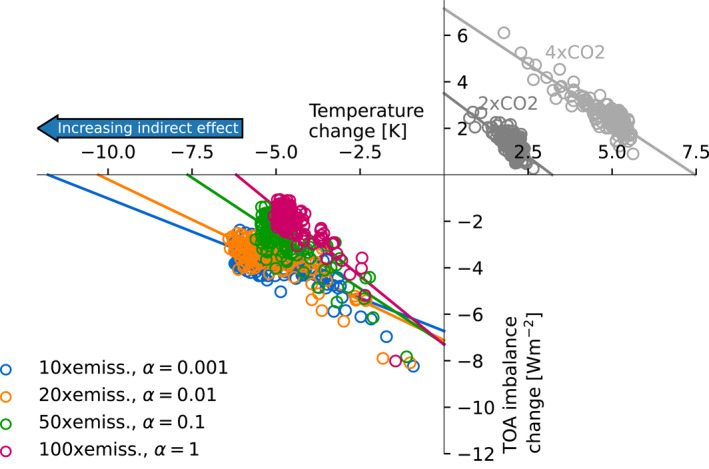
Gregory plot of four *abrupt‐aerosol* experiments with varying direct and indirect aerosol effect. The standard *abrupt‐2xCO2* and *abrupt‐4xCO2* simulations with MPI‐ESM1.2‐CR are shown for reference. The efficacy estimates for each case are presented in Table S1 in Supporting Information S1.

We first inspect four idealized experiments with similar forcing strength, achieved through different combinations of enhanced direct and indirect effects, as shown in Figure 2. The four experiments show a clear difference in their slopes, meaning that they have different values of the feedback parameter (*λ*). This indicates a difference in forcing efficacy, since the efficacy describes the magnitude of the feedback parameter compared to that of carbon dioxide (Equation 2). The feedback parameter is consistently smaller (less negative), and hence the efficacy is larger, for an enhanced indirect effect than for an enhanced direct effect. Thus, for a given effective radiative forcing the cooling is stronger when the ratio of indirect to direct aerosol cooling is large.

For the purpose of illustration the forcing in all four simulations is large compared to the present‐day aerosol cooling (Forster et al., 2021). This helps obtaining a clear signal: in weaker‐forcing scenarios the weak signal‐to‐noise ratio makes it difficult to distinguish the direct and indirect effect experiments from each other (see Figure S1 in Supporting Information S1). However, due to the state dependency of climate feedbacks (Bloch‐Johnson et al., 2015; Meraner et al., 2013), the value of the feedback parameter depends on the forcing strength. Therefore, we use six‐member ensembles to enable distinction between the direct and indirect effects also in cases with smaller forcing strength (around −2.2 to −3.2 Wm^−2^, see Figure S2 in Supporting Information S1), confirming that there is a difference in efficacy between the direct and indirect effect also in cases where the forcing strength is closer to the lower bound of estimates of the present‐day value (Forster et al., 2021).

### 4.2 Model Dependence

Next, we ask whether this behavior is model dependent, or if it is more likely to be a general behavior. There is some indication in the results of Richardson et al. (2019) that the aerosol forcing efficacy is larger in models which perturb aerosol emissions rather than concentrations. Models with perturbed emissions are typically also models that include an indirect effect through complex representations of aerosol‐cloud interactions, thus supporting the results obtained here.

To further investigate the model dependence we compared two versions of MPI‐ESM1.2 and found that in the LR version of the model the variation in the feedback parameter with the strength of the indirect effect persists, see Figure S3 in Supporting Information S1. The overall pattern is the same in both model versions, although the efficacy values in the corresponding cases are not the same (Table S1 in Supporting Information S1): the effect is larger in the CR model. The two versions of MPI‐ESM1.2 differ by more than resolution, most importantly, certain tuning parameters are not set the same way. The CR model version was finalized before the LR model version and uses a much smaller value of a parameter that enhances the amount of marine stratocumulus clouds. The large parameter value was set in MPI‐ESM1.2‐LR because it was found to induce a negative stratocumulus cloud feedback, and so dampened the very high climate sensitivity of MPI‐ESM1.2‐LR (Mauritsen & Roeckner, 2020). The MPI‐ESM1.2‐CR version instead has a weaker radiative forcing from CO_2_, which by chance results in a similar climate sensitivity to that of MPI‐ESM1.2‐LR. Our interpretation is that the negative stratocumulus cloud feedback in MPI‐ESM1.2‐LR dampens the remote region surface cooling, resulting in less sensitivity of the efficacy to aerosol indirect effects. Since the observational estimates of the stratocumulus cloud feedback is weak but positive (Myers et al., 2021) one could argue that the behavior of MPI‐ESM1.2‐CR in this regard is more realistic than that of the LR version.

In summary, within both model versions we have used, the cooling from an enhanced indirect effect is larger than the cooling from the direct effect. We find anecdotal evidence for this also in other models, and therefore there is strong model based evidence that enhancing the indirect effect causes a larger forcing efficacy.

### 4.3 Spatial Distribution of Forcing and Temperature Change

To identify the mechanisms behind the larger efficacy of the aerosol indirect effect, we study the spatial patterns of forcing and resulting temperature change. As described in the following, we find that the indirect effect causes a remote temperature change at mid‐ to high‐latitudes, in contrast to the local tropical response to the direct effect. In addition, we find that variations in the latitude of the forcing seem to be of importance to the magnitude of the temperature response.

**Figure 3:**
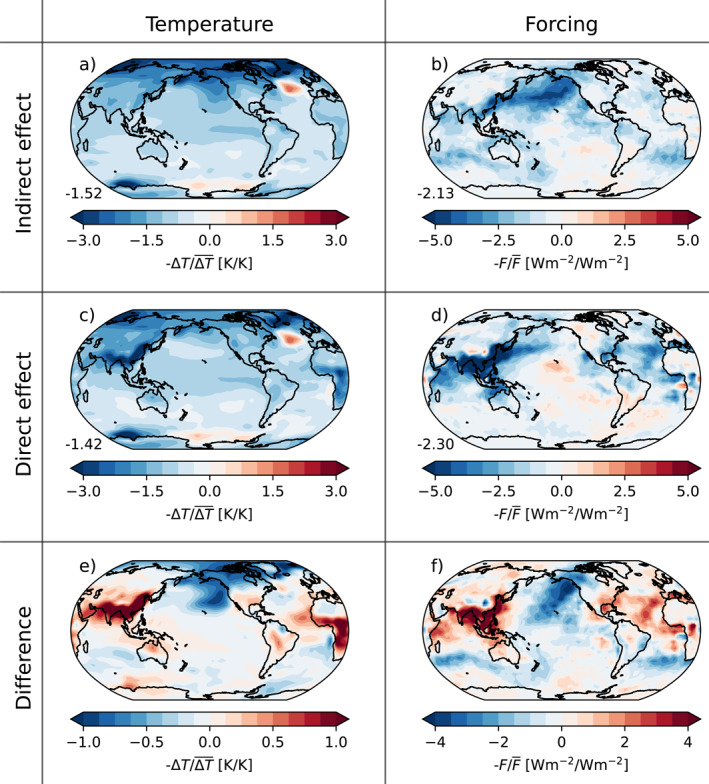
Maps showing the temperature anomaly (Δ*T*, first column) and forcing (*F*, second column) averaged over the last 30 years of *abrupt‐aerosol* experiments with enhanced indirect effect (a–b) and direct effect (c–d), as averaged over six‐member ensembles. Panels (e–f) show the difference in temperature and forcing between the two experiments. All values are normalized against the global average to better show the spatial pattern, and multiplied by − 1 to get a more intuitive color scale (darker blue signifies stronger cooling than the global average). The forcing was obtained from fixed‐SST simulations while the temperature changes are from fully coupled simulations. The numbers in the lower left corner in panels (a–d) show the global mean, in K and Wm^−2^, respectively. The parameter values are *1xemiss.,* *α* = 0.01 (indirect effect) and *5xemiss.,* *α* = 1 (direct effect).

First, we examine the temperature change and forcing in two experiments with similar global mean forcing strength (*1xemiss.*, *α* = 0.01 and *5xemiss.*, *α* = 1, Figure 3). The aerosol indirect effect causes a forcing mainly over the North Pacific, likely because aerosols emitted in South and East Asia, which are the regions with the heaviest emissions in 2005 (Stevens et al., 2017), are assumed to be transported with the westerlies over the ocean (Figure 3b). Above the ocean the optical depth of the aerosols is initially small, so enhancing the indirect effect has a large effect there, consistent with the mechanism of the Twomey effect (Carslaw et al., 2013). When the background aerosol optical depth scaling factor (*α*) is reduced the indirect effect becomes stronger, causing strong forcing in the remote regions. The forcing drives a local cooling, as well as a remote temperature response over the Arctic (Figure 3a). In contrast, enhancing the direct effect gives a forcing as well as a temperature response that are localized to major emission source regions, mainly in South and East Asia and Central Africa (Figures 3c and 3d).

The radiative forcing from the indirect effect is concentrated over the northern part of the Pacific Ocean (Figure 3b). Dong et al. (2019) showed that local SST changes in that area have little to no effect on the global average net TOA radiation balance. This means that the global mean temperature response per unit forcing will be larger for a forcing there than in the case of a forcing in the equatorial West Pacific where an SST change is efficiently propagated throughout the troposphere and hence dampened by negative feedbacks. Since Dong et al. (2019) forced the system with warmed SSTs they did not chart the effect of a local temperature change over land, but it seems reasonable to expect a similar response to changes over South and East Asian land areas as to changes over the northern Indian Ocean and the tropical West Pacific Ocean. Therefore, the direct effect, with forcing over land in Asia, results in a stronger change to the TOA radiation balance and thus a small efficacy. Furthermore, the forcing is located at different latitudes in the two cases, with the indirect effect causing cooling preferentially at higher latitudes (Figure 3). A connection between extratropical forcing and a large aerosol forcing efficacy is in line with previous studies (e.g., Salvi et al., 2022), and also supported by studies on the latitude dependence of other forcing agents (e.g., Hansen et al., 1997; Hansen et al., 2005; Stuecker et al., 2020). Thus, based on the current understanding of physical processes and previous studies we argue for the general validity of our conclusions.

### 4.4 Emissions From Single Source Regions

**Figure 4:**
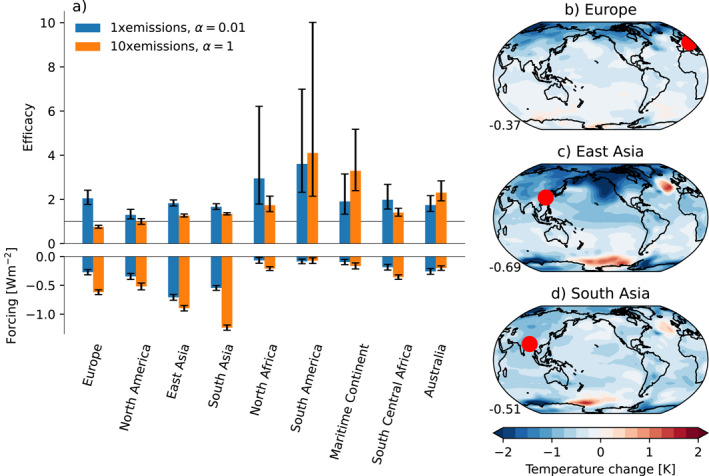
(a) Forcing efficacy (Equation 3) (upper part) and global mean radiative forcing (lower part) in simulations with emissions from each region separately, and (b–d) the spatial pattern of temperature change from emissions in the three regions that cause the largest global mean temperature change, with *α* = 0.01. Error bars in panel (a) show the 68% range and were obtained using Monte Carlo sampling for the efficacy, and the standard deviation of the mean in the final 20 years of fixed‐SST simulations for the forcing. Red dots on the maps indicate emission source locations.

The latitude dependence can be further investigated in simulations with emissions from one source location at a time. Figure 4a shows the forcing efficacy in simulations with emissions from each of the nine source regions in MACv2‐SP. In the cases that show a signal that is distinguishable from the noise, the efficacy of an enhanced indirect effect is consistently larger than that of an enhanced direct effect (in Europe, East and South Asia, and, to some extent, North America). Emissions from Europe stand out with a very large efficacy from aerosol‐cloud interactions.

The patterns of temperature change resulting from an enhanced indirect effect in the three cases with the strongest emissions (Europe and South and East Asia) are shown in Figures 4b–4d. The forcing and temperature change from all emission source regions with enhanced direct and indirect effect, respectively, are shown in Figures S4–S7 in Supporting Information S1. The results show a strong Arctic response from both the European and East Asian emissions. However, whereas the global mean forcing is the strongest from East and South Asian emissions, European emissions contribute disproportionally to the global mean temperature change due to the strong cooling in the Arctic. The enhanced Arctic response suggests that mechanisms related to the Arctic amplification or ocean energy transport (e.g., Acosta Navarro et al., 2016; Pithan & Mauritsen, 2014) are likely to dominate the temperature response to European aerosol emissions.

There are large statistical uncertainties in the efficacy in some of the regions (North Africa, South America, the Maritime Continent, South Central Africa, and Australia). In those regions the emissions in 2005, and thus also the forcing, are weak (Figure 4a). Therefore, the signal is obscured by the internal variability of the climate system and no conclusion can be drawn regarding the forcing efficacy from emissions in those regions.

## 5 Conclusions and Implications

We have shown that in MPI‐ESM1.2, aerosol forcing from an enhanced aerosol indirect effect causes a temperature response per unit forcing that is larger than the corresponding response to forcing from increased aerosol emissions. In other words, the aerosol forcing efficacy is larger when the ratio of indirect to direct effect is large. The response to the enhanced indirect effect is dominated by remote oceans and an Arctic‐amplified cooling, in contrast to the direct effect which causes a radiative forcing and a resulting temperature response localized to major emission source regions. Indirect effects from European emissions contribute disproportionately to the strong Arctic cooling, while the overall stronger emissions in South and East Asia dominate the total response.

We provide a mechanistic explanation for the enhanced remote response to the aerosol indirect effect. An enhanced indirect effect induces stronger forcing in mid‐ to high‐latitude remote ocean regions where the aerosol optical depth is low to begin with, and a forcing in the mid‐ and high latitudes generally leads to a larger forcing efficacy compared to a forcing closer to the equator (e.g., Hansen et al., 1997; Hansen et al., 2005; Salvi et al., 2022; Stuecker et al., 2020).

A larger‐than‐unit aerosol forcing efficacy reported in recent studies (Salvi et al., 2022; Smith & Forster, 2021) could be related to a large efficacy of the aerosol indirect effect in the models applied in those studies. Furthermore, our results could help reconcile intermodel differences in the temperature response to aerosol forcing (e.g., Richardson et al., 2019). A larger aerosol forcing efficacy also has implications for estimates of the climate sensitivity based on the historical warming (e.g., Otto et al., 2013), and projections of future aerosol forcing when emissions from fossil fuel burning eventually decline.

## Supporting information

Supporting Information S1

## Data Availability

The source code for MPI‐ESM1.2 is available through https://mpimet.mpg.de/en/science/models/mpi-esm (Mauritsen et al., 2019). The output data used to produce the figures for this paper, and the accompanying Python scripts, are available through Zenodo at https://doi.org/10.5281/zenodo.7198850 (Huusko et al., 2022). The observational temperature data presented in Figure 1 was downloaded via https://crudata.uea.ac.uk/cru/data/temperature/ (Morice et al., 2021).
