# Supplementary material for: Stronger Response to the Aerosol Indirect Effect Due To Cooling in Remote Regions
Source: Geophys Res Lett. 2022 Oct 31;49(21):e2022GL101184. doi: 10.1029/2022GL101184 (PMC9788190; doi:10.1029/2022GL101184)
Supplement: Supplementary file 1 — Supporting Information S1 [file GRL-49-e2022GL101184-s001.pdf]

Geophysical Research Letters

Supporting Information for

Stronger Response to the Aerosol Indirect Effect due to Cooling in Remote Regions

Linnea Huusko[1], Angshuman Modak[1*], Thorsten Mauritsen[1]

[1]Department of Meteorology, Stockholm University, Stockholm, Sweden

Contents of this file

Figures S1 to S7
Table S1

Introduction

Figure S1 shows the feedback parameter as a function of the forcing strength in a set of abrupt-aerosol simulations with varying emission strength and strength of the aerosol indirect effect. Simulations were run with emissions and Twomey effect ranging from the model standard to strongly enhanced. The figure indicates that the feedback parameter is smaller (less negative) for a larger ratio of indirect to direct efßfect.

Figure S2 displays how the use of ensemble averaging reduces the noise in the data. The bottommost panel confirms that the feedback parameter is smaller in the cases with an enhanced indirect effect.

Figure S3 corresponds to Figure 2 but the simulations have been run in the LR version of MPI-ESM1.2. Note that in the LR model the four simulations do not have the same forcing strength. Instead they intersect at a similar temperature change. This may give the impression that the pattern in Figure S3 is opposite of that in Figure 2, which is not the case: had the forcing strengths lined up the two figures would look more similar. An estimate of the efficacy in all simulations in Figures 2 and S3 are shown in Table S1.

*Now at Interdisciplinary Programme in Climate Studies, Indian Institute of Technology Bombay, Mumbai, India

Figures S4 and S5 show the temperature change with enhanced indirect and direct effects, respectively, with emissions isolated to each of the nine source regions in MACv2-SP. Figures S6 and S7 show the corresponding radiative forcing.

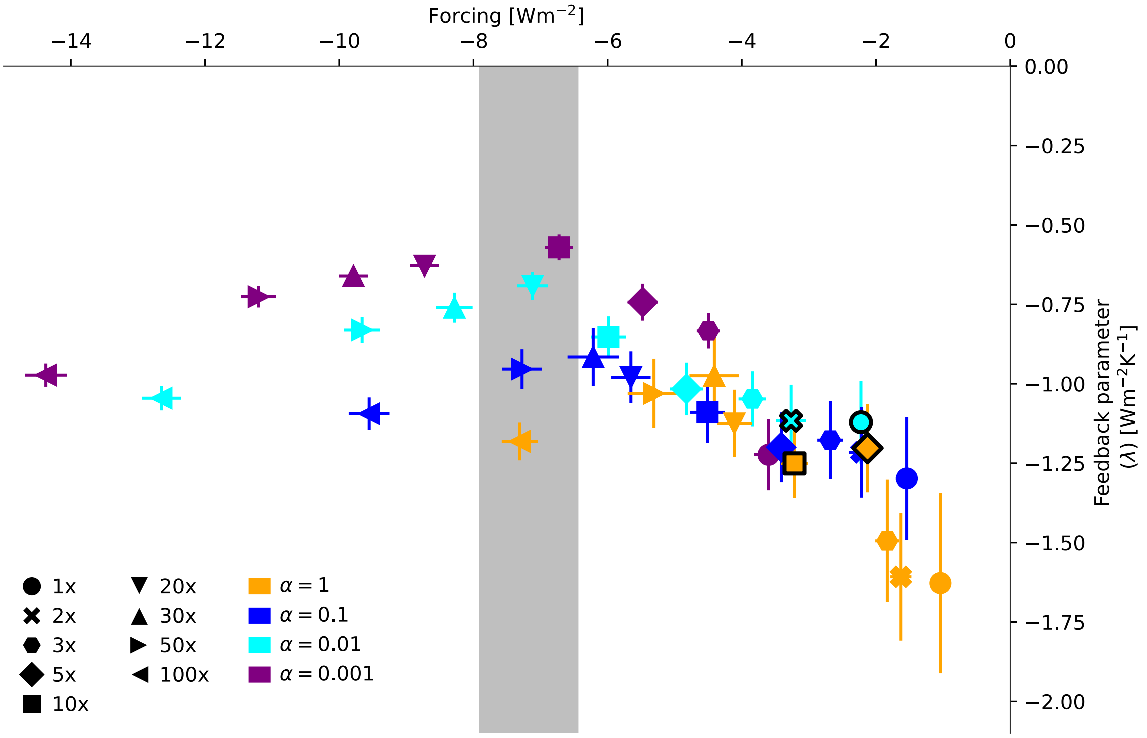

Figure S1. The feedback parameter as a function of the radiative forcing in *abrupt-aerosol* simulations with different combinations of the emission strength (different symbols) and values of the background aerosol scaling parameter (α, different colors). Gregory plots for the four points in the grey box are shown in Figure 2. The four points marked with a black outline are examined further in Figure S2. Error bars show standard errors from the linear regression.

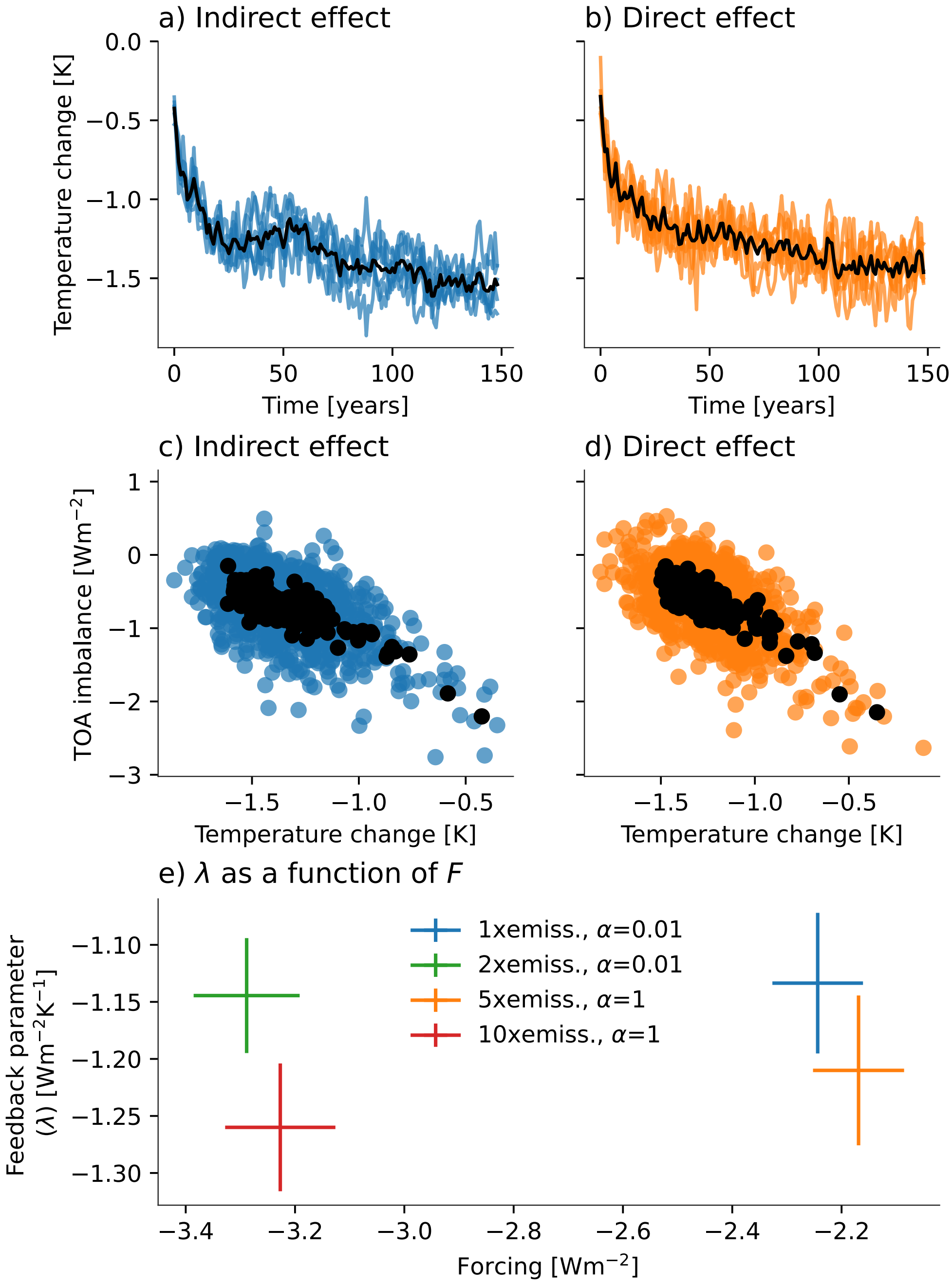

Figure S2. Comparison of two *abrupt-aerosol* experiments (with *1xemissions*, $\alpha = 0.01$ and *5xemissions*, $\alpha = 1$): evolution of temperature change with time (a-b) and Gregory plots (TOA imbalance against temperature change, c-d). Lines and dots in colour show individual ensemble members while black lines and dots show the six-member ensemble average. Panel e shows the feedback parameter as a function of forcing strength in the four experiments marked with black outlines in Figure S1. The error bars show the standard error from linear regression.

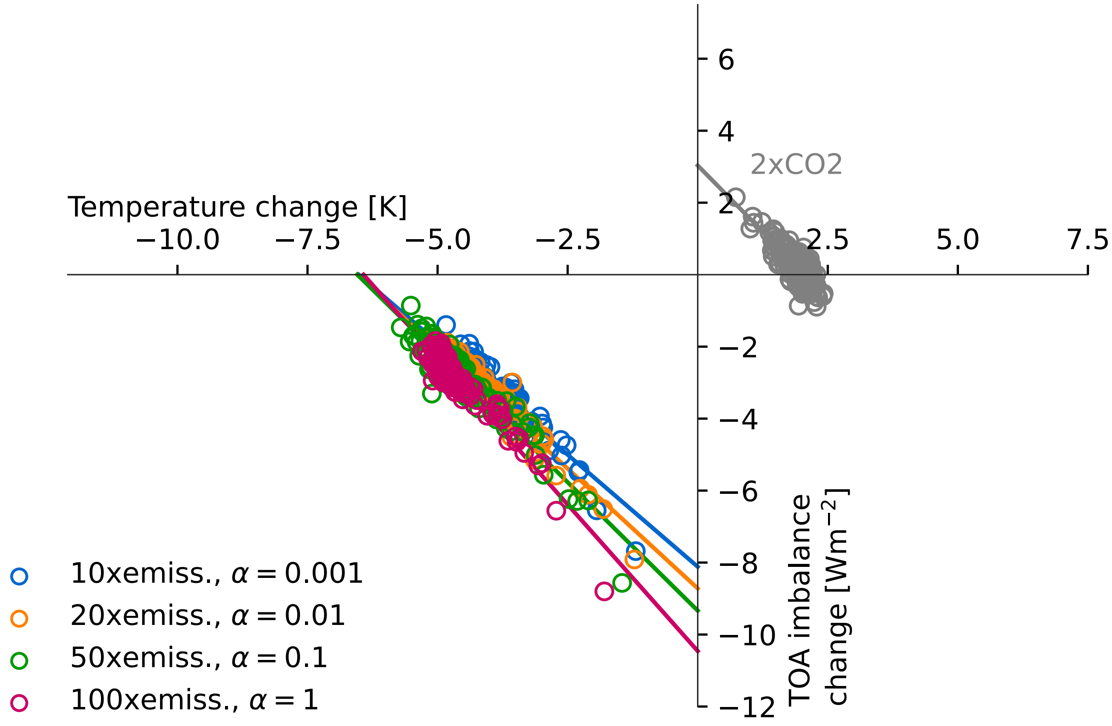

Figure S3. As Figure 2 but simulations run in the LR (low resolution) version of MPI-ESM1.2.

Table S1. Efficacy (E) values in the simulations shown in Figures 2 and S3, calculated using equation (2). The feedback parameter for $2xCO_2$ is -1.09 Wm^2K^{-1} in the CR model and -1.47 Wm^2K^{-1} in the LR model.

	10xemiss. $\alpha = 0.001$	20xemiss. $\alpha = 0.01$	50xemiss. $\alpha = 0.1$	100xemis. $\alpha = 1$
CR	1.91	1.58	1.14	0.92
LR	1.18	1.10	1.03	0.90

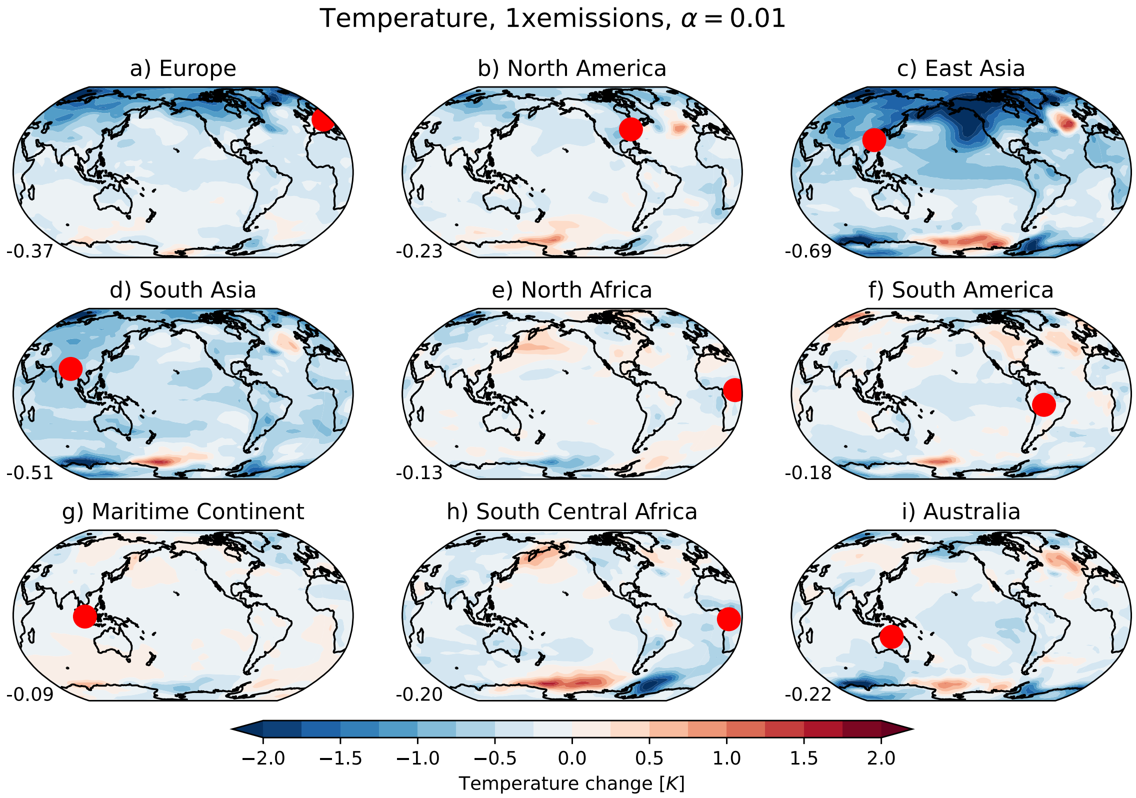

Figure S4. Maps of the absolute temperature change averaged over the last 30 years of 150 year long *single-plume* experiments with *1xemissions* and $\alpha = 0.01$. Each panel shows the resulting temperature pattern when the model is forced by emissions from a single plume only (emissions from all other plumes held at zero). The red dot on each map shows the plume location. The number in the lower left corner of each panel is the global mean temperature change in K.

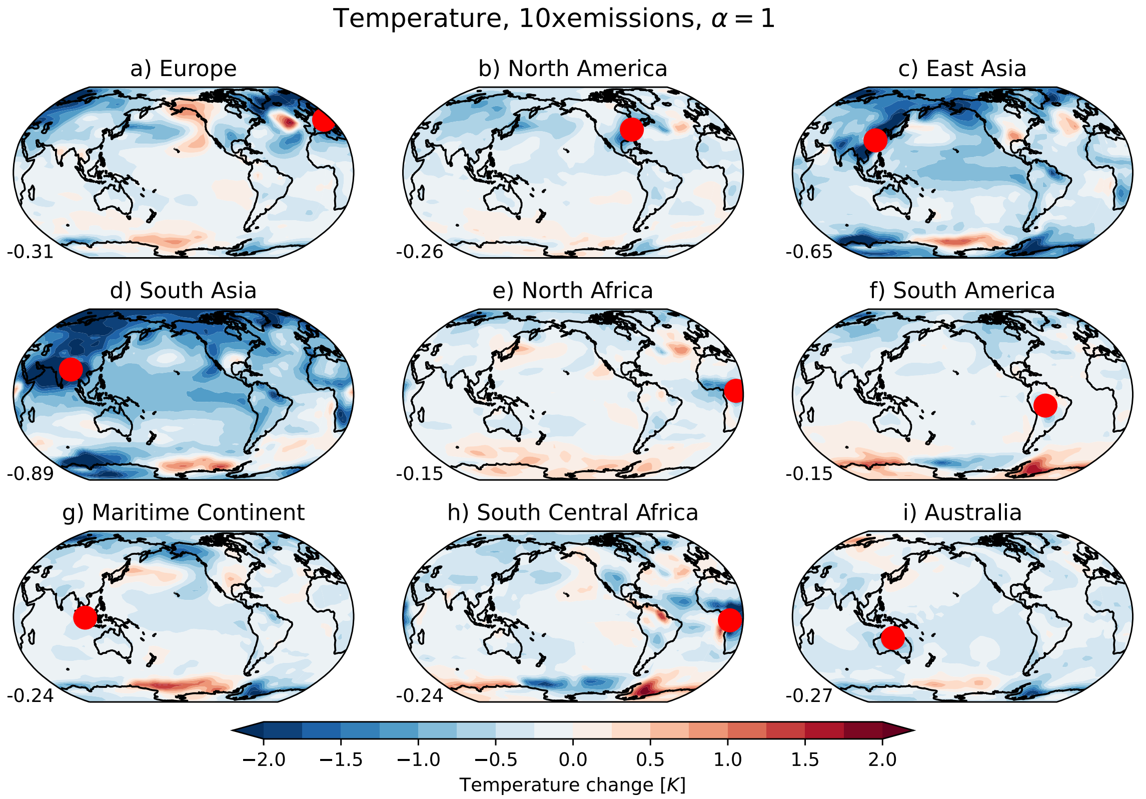

Figure S5. As Figure S4 but for experiments with *10xemissions* and $\alpha = 1$. The number in the lower left corner of each panel is the global mean temperature change in K.

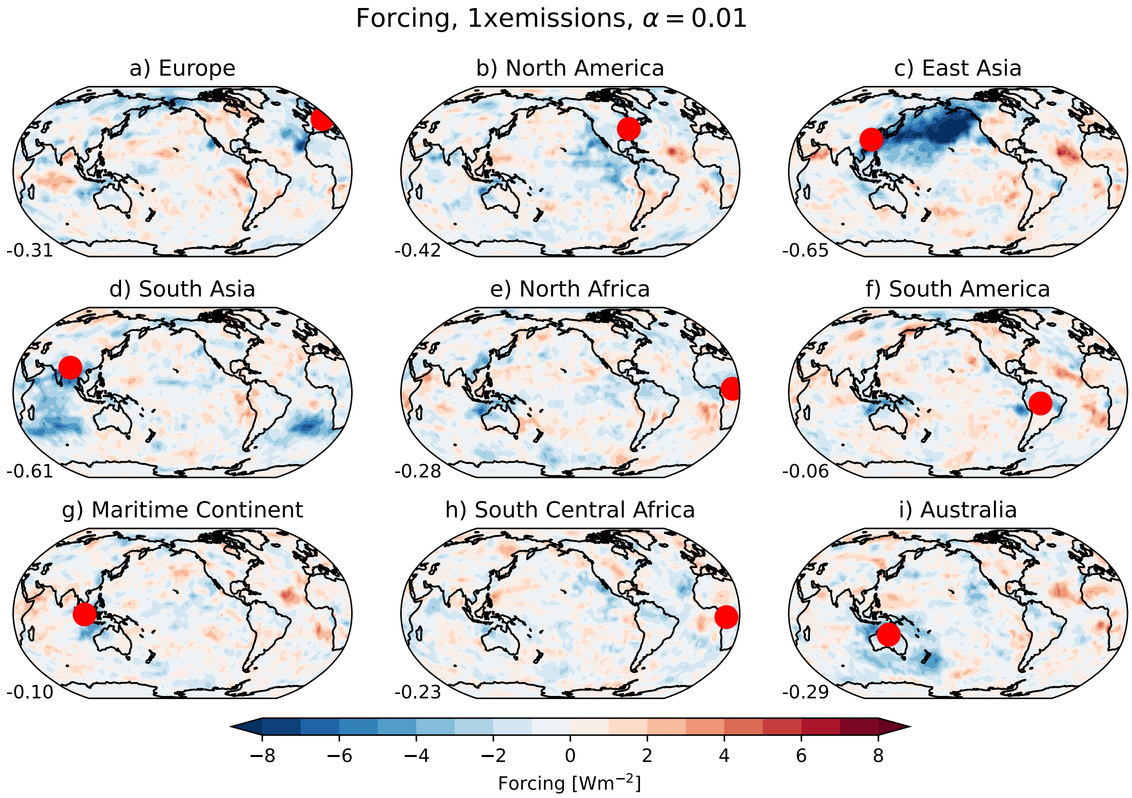

Figure S6. As Figure S4 but for forcing, averaged over the last 20 years of the 30 year long simulations. The number in the lower left corner of each panel is the global mean forcing in Wm^{-2}.

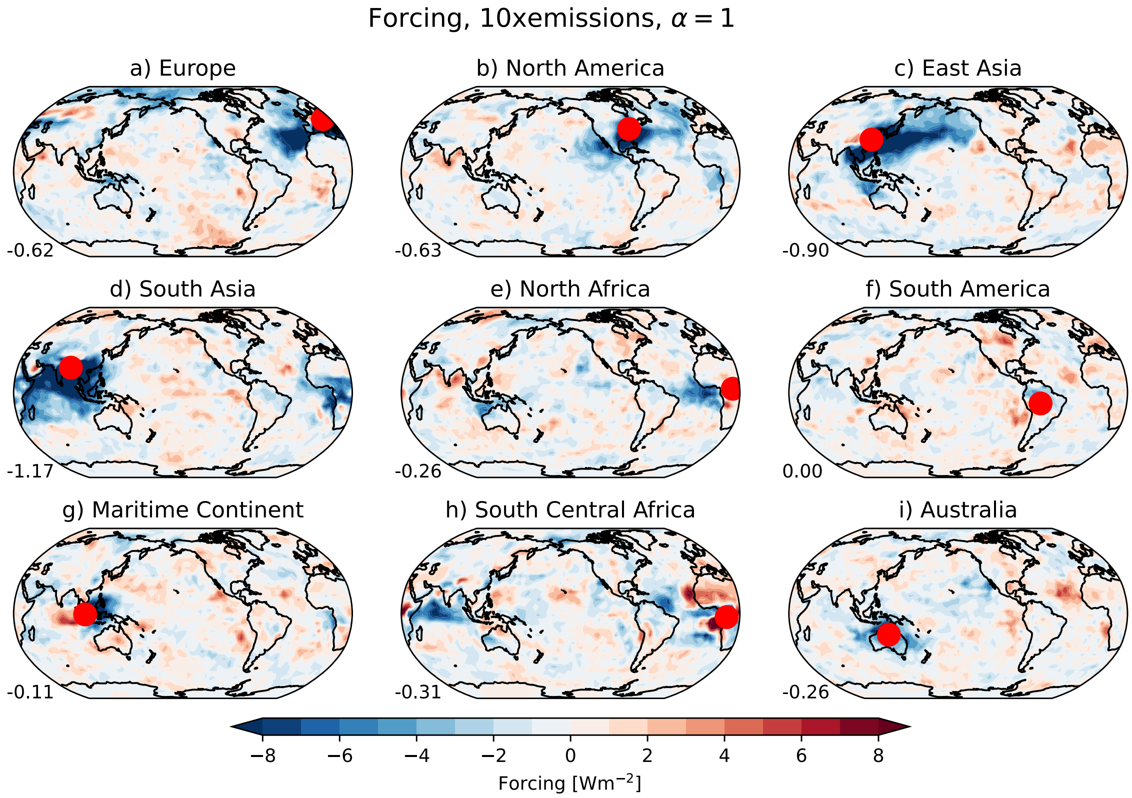

Figure S7. As Figure S5 but for forcing, averaged over the last 20 years of the 30 year long simulations. The number in the lower left corner of each panel is the global mean forcing in Wm^{-2}.
